# Novel Anti-Adhesive CMC-PE Hydrogel Significantly Enhanced Morphological and Physiological Recovery after Surgical Decompression in an Animal Model of Entrapment Neuropathy

**DOI:** 10.1371/journal.pone.0164572

**Published:** 2016-10-14

**Authors:** Hideki Urano, Katsuyuki Iwatsuki, Michiro Yamamoto, Tetsuro Ohnisi, Shigeru Kurimoto, Nobuyuki Endo, Hitoshi Hirata

**Affiliations:** 1 Department of Orthopaedic Surgery, Yokkaichi municipal hospital, 2-2-37 Shibata-cho, Yokkaichi, Mie, 510–8567, Japan; 2 Department of Hand Surgery, Nagoya University Graduate School of Medicine, 65 Tsurumai-cho, Showa-ku, Nagoya, Aichi, 466–8550, Japan; 3 Healthcare Business Development, Teijin Limited, 4-3-2 Asahigaoka Hino, Tokyo, 191–8512, Japan; Szegedi Tudomanyegyetem, HUNGARY

## Abstract

We developed a novel hydrogel derived from sodium carboxymethylcellulose (CMC) in which phosphatidylethanolamine (PE) was introduced into the carboxyl groups of CMC to prevent perineural adhesions. This hydrogel has previously shown excellent anti-adhesive effects even after aggressive internal neurolysis in a rat model. Here, we confirmed the effects of the hydrogel on morphological and physiological recovery after nerve decompression. We prepared a rat model of chronic sciatic nerve compression using silicone tubing. Morphological and physiological recovery was confirmed at one, two, and three months after nerve decompression by assessing motor conduction velocity (MCV), the wet weight of the tibialis anterior muscle and morphometric evaluations of nerves. Electrophysiology showed significantly quicker recovery in the CMC-PE group than in the control group (24.0 ± 3.1 vs. 21.0± 2.1 m/s (p < 0.05) at one months and MCV continued to be significantly faster thereafter. Wet muscle weight at one month significantly differed between the CMC-PE (BW) and control groups (0.148 ± 0.020 vs. 0.108 ± 0.019%BW). The mean wet muscle weight was constantly higher in the CMC-PE group than in the control group throughout the experimental period. The axon area at one month was twice as large in the CMC-PE group compared with the control group (24.1 ± 17.3 vs. 12.3 ± 9 μm^2^) due to the higher ratio of axons with a larger diameter. Although the trend continued throughout the experimental period, the difference decreased after two months and was not statistically significant at three months. Although anti-adhesives can reduce adhesion after nerve injury, their effects on morphological and physiological recovery after surgical decompression of chronic entrapment neuropathy have not been investigated in detail. The present study showed that the new anti-adhesive CMC-PE gel can accelerate morphological and physiological recovery of nerves after decompression surgery.

## Introduction

Postoperative adhesions and perineural scarring are major causes of failure after peripheral nerve surgery [1,2]. Intraoperative nerve damage, bleeding in the operating field, or even simple manipulation of a nerve can cause adhesive scarring [3]. Reportedly, 1% to 25% of patients who undergo carpal tunnel release develop symptoms related to residual scar tissue [2,4]. Surrounding tissue adhering to the median nerve can also lead to recurrent carpal tunnel syndrome (CTS), which is associated with an extremely high re-recurrence rate [5].

Many surgical techniques have been developed to prevent perineural adhesions, including vein wrapping, muscle flaps and free fat grafts [5,6,7,8,9,10]. However, a specific technique has not yet been standardized. We recently developed a novel hydrogel to prevent perineural adhesions, derived from sodium carboxymethylcellulose (CMC) in which phosphatidylethanolamine (PE) was introduced into the carboxyl groups of CMC. The anti-adhesive effect of the hydrogel is excellent even after aggressive internal neurolysis in a rat model [4].

The present study tests the hypothesis that CMC-PE hydrogel is useful in the context of not only inhibiting adhesion but also of the enhancement of morphological and physiological recovery after surgery for chronic compression nerve damage in a disease model in vivo.

## Materials and Methods

The Animal Ethics Research Committee of Nagoya University approved all experimental and animal maintenance protocols (Permit No: 25154), which proceeded in accordance with the Animal Protection and Management Laws of Japan (No. 105) and the Ethical Issues of the International Association for the Study of Pain.

### Animal model

**Fig 1:**
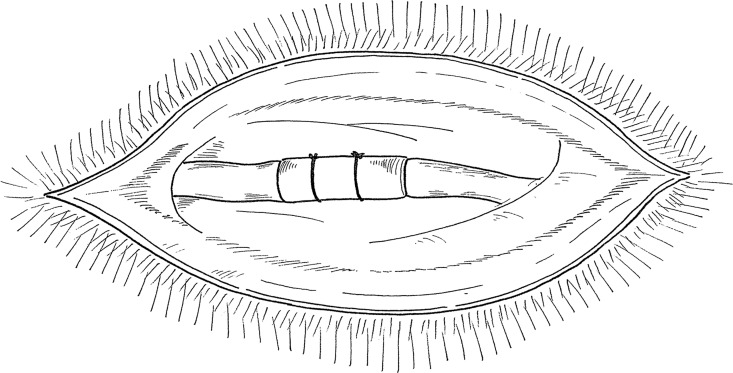
Silicone tubing (length, 10 mm; internal diameter, 1.3 mm) wrapped around sciatic nerve is secured by two 5–0 ethilon sutures.

Male Lewis rats (n = 63; body weight, ~250 g) were anesthetized with an intraperitoneal injection of 5% pentobarbital and then assigned to the following groups. One group received only a skin incision (sham), while chronic nerve compression was created in the control and CMC-PE groups (n = 21 per group) as described [11,12,13,14]. Briefly, the right sciatic nerve was exposed at the level of the mid-thigh and a longitudinally incised silicone tube (length, 10 mm; internal diameter, 1.3 mm) was wrapped around the nerve. Two 5–0 ethilon sutures were wrapped around the tube to prevent it from becoming dislodged Fig 1. Three months after the first operation, all rats underwent a second operation. The sham group received only a second skin incision. The silicone tube was removed from the control and CMC-PE groups to decompress the nerve and the wound was closed in the control group without any adjuvant treatment. The CMC-PE group was treated with 0.5 mL of 1.0 wt% CMC-PE hydrogel as described before wound closure [4]. Sodium CMC (Nippon Paper Chemicals, Tokyo, Japan) was dissolved in water and stirred overnight. Tetrahydrofuran (THF) (Wako Pure Chemicals, Tokyo, Japan) was then added dropwise over 30 min. To this solution, dioleoyl PE (NOF, Tokyo) was added. A solution of 1-ethyl-3-[3-(dimethylamino)-propyl]-carbobiimide hydrochloride (Osaka Synthetic Chemi- cal Labs, Osaka, Japan) and 1-hydroxybenzotriazole monohydrate (Osaka Synthetic Chemical Labs) in THF/water (1:1) was then added dropwise over 60 min, during which the pH of the solution was maintained at 6.8 by the addition of 0.1 M NaOH, and the reaction was allowed to proceed overnight. After evaporation of the organic solvent, the product was purified by ethanol precipitation and vacuum-dried to yield CMC–PE as a white powder.The powder was sterilized using ethylene oxide gas and dissolved in sterilized water under aseptic conditions. We prepared high-viscosity CMC-PE hydrogel (viscosity, 306 P, 1.0 wt.%). CMC–PE hydrogel is a physically crosslinked hydrogel. In water, hydrophobic association occurs among the phospholipids bound to CMC, and the CMC–PE hydrogel becomes viscous.

**Fig 2:**
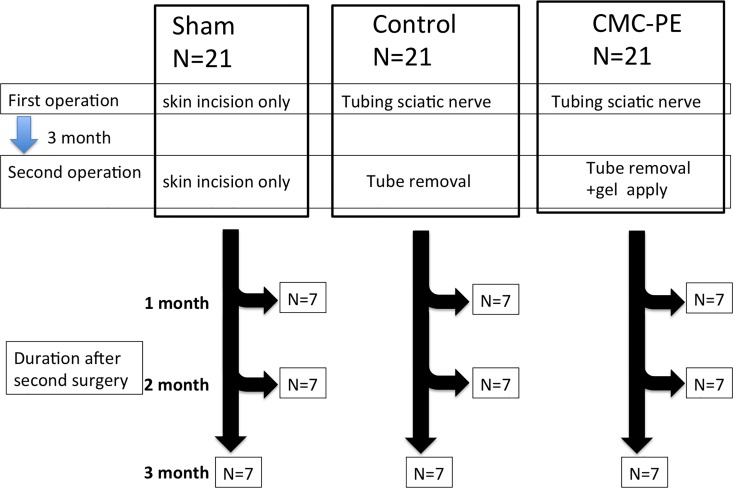
Six rats out of 7 were used for Electrophysiological and Biomechanical analysis. 1 rat out of 7 was used for Morphometric analysis in each subgroups.

The rats were then maintained for up to three months according to the approved procedure for evaluating functional recovery. Fig 2

### Electrophysiological evaluation

Under anesthesia, the rats were electrophysiologically assessed immediately after and at one, two and three months after nerve decompression to measure the motor conduction velocity (MCV) in 63 tibial nerves (n = 6 per month per group).

Compound muscle action potentials (CMAPs) in exposed tibialis anterior muscle were measured at room temperature (24°C) by placing two H537A stainless steel monopolar recording electrodes (Nihon Kohden, Tokyo, Japan) on the center of the belly of the muscle. The sciatic nerve was exposed and a UM2-5050 bipolar stimulating electrode (Nihon Kohden) was placed bilaterally around the nerve at the level of the sciatic notch. Supramaximal electrical stimulation was applied for 100 ms at a frequency of 1 Hz; square wave using an SS-201J isolator (Nihon Kohden) connected to an electronic stimulator. The MCV was calculated as described [15,16].

### Biomechanical analysis

**Fig 3:**
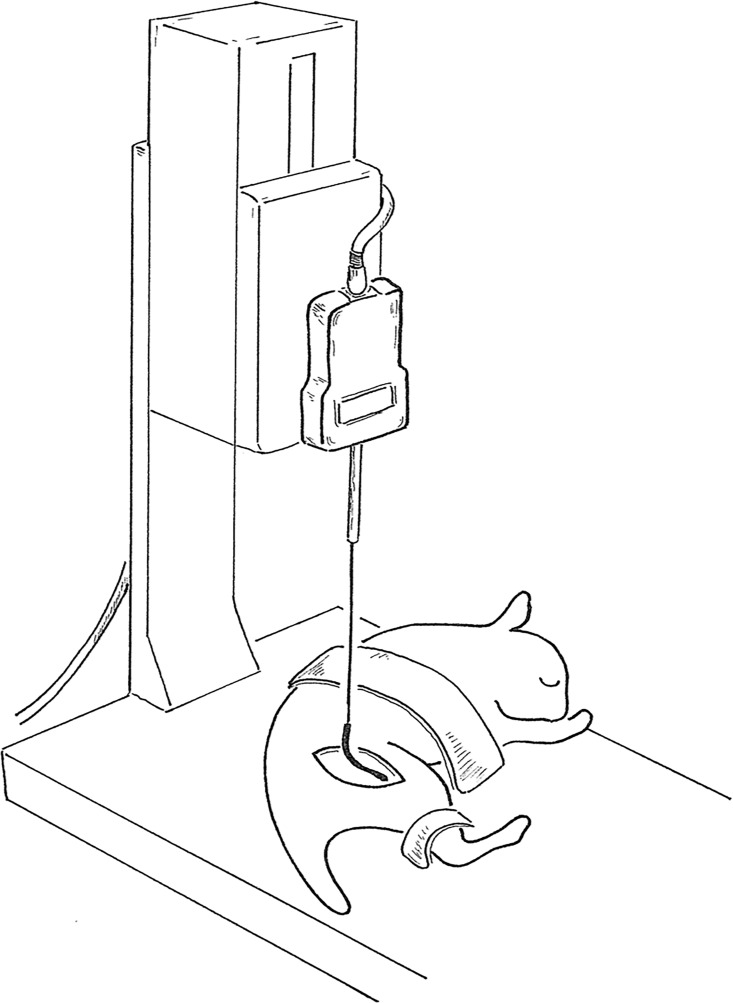
Biomechanical testing. Proximal stump mounted on digital force gauge. Traction was applied to nerve until it completely detached from neural bed and ultimate strength was recorded.

The ultimate breaking strength of nerve adhesion was biomechanically assessed after the electrophysiological tests in 54 nerves (n = 6 per month per group) as described [4,17]. Briefly, sciatic nerves were transected 5 mm proximal to the compression site, the proximal stump was mounted on an FGP-0.2 digital force gauge (Shimpo, Kyoto, Japan) and the operated limb was fixed to the stage on the gauge. The proximal end was ligated using a suture connected to the load cell Fig 3. A rate of 2 cm/min was applied to the nerve until it completely detached from the neural bed and the ultimate strength was recorded.

### Wet muscle weight

Fifty-four (n = 6 per month per group) tibialis anterior muscles of euthanized rats were dissected free from their origins and insertions and immediately weighed. The relative wet weight of the tibialis anterior muscle is expressed as a ratio (%) of total body weight as described [16,18].

### Morphometric analysis

Nerves were fixed in phosphate-buffered 4.0% glutaraldehyde for 24 h, embedded in Epon and cut into 0.25-μm-thick slices. The specimens were then stained with toluidine blue and axons were morphometrically analyzed using computer-assisted image analysis. Briefly, histological features of nerve fibers were quantified using an FSX100 microscope (Olympus, Tokyo, Japan) equipped with a charge-coupled device video camera, Photoshop (Adobe, San Jose, CA, USA), and ImageJ software (US National Institutes of Health, Bethesda, MD, USA).

**Fig 4:**
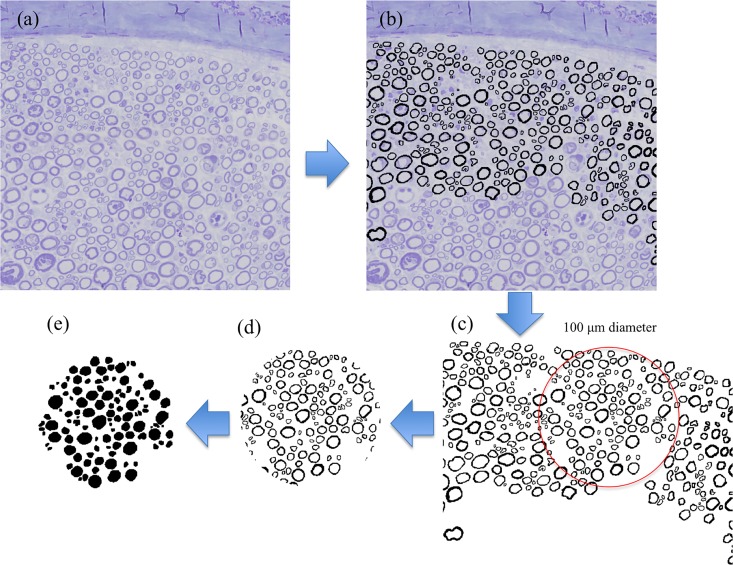
Histological assessment of nerve specimens. Nerve stained with toluidine blue (a). Trace of axon (b) and axonal areas included in circle (diameter, 100 μm) below perineurium (c, d, e).

Morphological changes such as axon stenosis and demyelination in nerve fibers with entrapment neuropathies tend to be more advanced at the outer portions of nerve fascicles [19]. Therefore, a research assistant who was blinded to the groups randomly selected five circular regions of interest (ROIs) with a diameter of 100 μm in the periphery of nerve fascicles. Axonal counts and the average axon area were evaluated using ImageJ Fig 4.

### Statistical analysis

All data were expressed as mean ± standard deviation (SD).

Data were statistically analyzed using SPSS software (SPSS Inc., Chicago, IL, USA). Differences among three groups were analyzed using a one-way analysis of variance (ANOVA), and the Tukey test was applied post hoc when significance was determined by the ANOVA. Differences between two groups were assessed using Student’s t-test. Values of p < 0.05 were considered statistically significant.

## Results

### Electrophysiological evaluation

**Fig 5:**
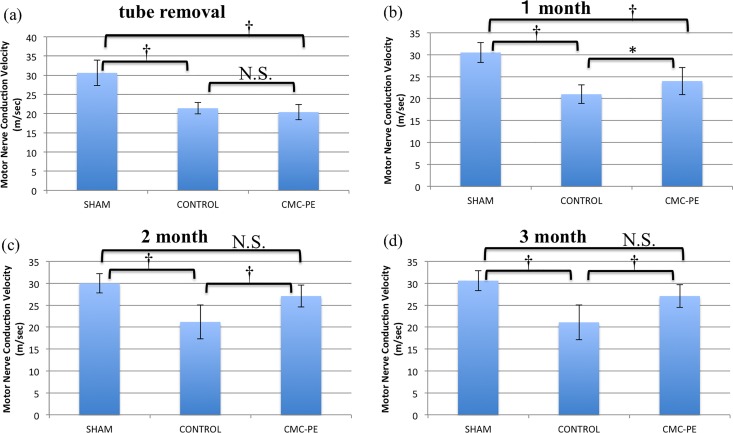
Electrophysiological testing after nerve decompression. At tube removal (a) and one month after decompression (b). Significant differences at one(b), two (c) and three months (d). *P < 0.05, †P < 0.01 (n = 6).

Fig 5A shows that MCV immediately after tube removal did not differ between the control and CMC-PE groups. Fig 5B–5D show serial changes in MCV after removing the silicone tube. The MCVs in the sham, control and CMC-PE groups at one, two and three months after nerve decompression were 30.5 ± 2.3, 21.0± 2.1 and 24.0 ± 3.1, 30.0 ± 2.2, 21.2 ± 3.9 and 27.1 ± 2.5 and 30.6 ± 2.3, 21.1 ± 4.0 and 27.1 ± 2.6 m/s, respectively. The MCV significantly differed between the control and CMC-PE groups at one month. And this trend continued to the end of study.

### Biomechanical analysis

**Fig 6:**
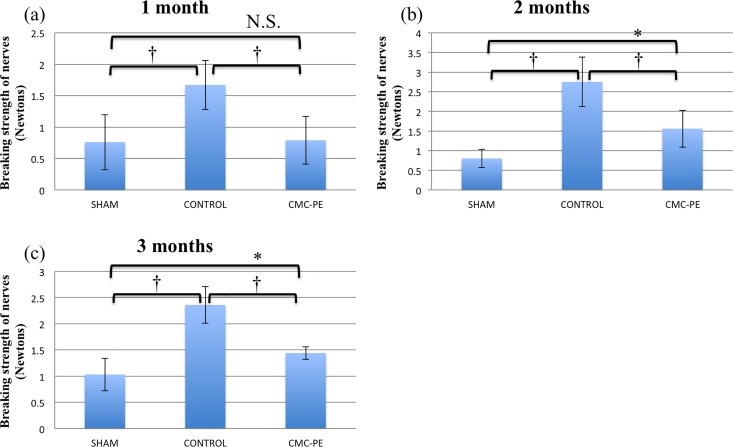
Serial changes in ultimate breaking strength of nerve adhesion after decompression at one (a), two (b) and three (c) months. *P < 0.05, †P < 0.01 (n = 6).

Fig 6 shows the ultimate breaking strength of nerve adhesion at each time point. The values for ultimate breaking strength in the sham, control and CMC-PE groups at one, two and three months after nerve decompression surgery were 0.76 ± 0.44, 1.67 ± 0.39 and 0.79 ± 0.38, 0.80 ± 0.23, 2.75 ± 0.63 and 1.56 ± 0.47 and 1.03 ± 0.31, 2.36 ± 0.35 and 1.44 ± 0.12N, respectively. The ultimate breaking strength of nerve adhesion remained significantly lower for the CMC-PE group than the control group at all time points, and tended to be even lower than that in the sham group at one month. These results indicated the powerful and persistent anti-adhesive effects of the CMC-PE hydrogel.

### Wet muscle weight

**Fig 7:**
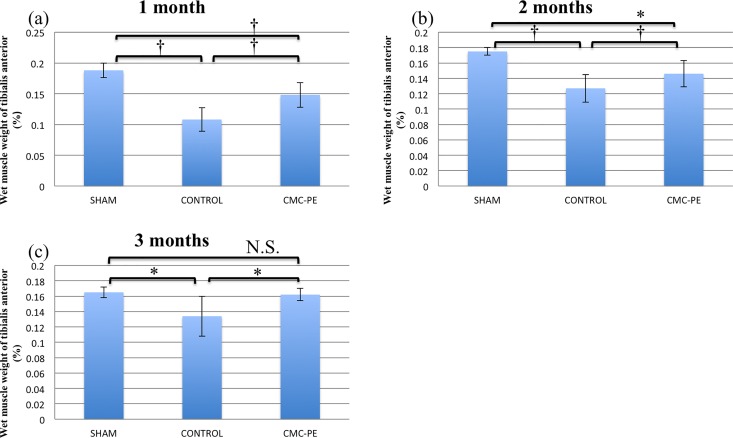
Mean wet muscle weights after nerve decompression. Mean wet muscle weight was constantly higher in the CMC-PE group than in the control group throughout experimental period. Significant differences at one (a) and three (c), but not at two months (b). *P < 0.05, †P< 0.01 (n = 6)

Fig 7 shows serial changes in the relative wet weight of the tibialis anterior muscle after nerve decompression. These values in the sham, control and CMC-PE groups at one, two and three months after nerve decompression surgery were 0.188 ± 0.012, 0.108 ± 0.019 and 0.148 ± 0.020, 0.175 ± 0.005, 0.127 ± 0.018 and 0.146 ± 0.017 and 0.165 ± 0.007, 0.134 ± 0.026 and 0.162 ± 0.008%, respectively. The tibialis anterior muscle was constantly heavier in the CMC-PE group than in the control group. These findings imply that muscle function recovered faster in the CMC-PE group than in the control group.

### Morphometric analysis

**Fig 8:**
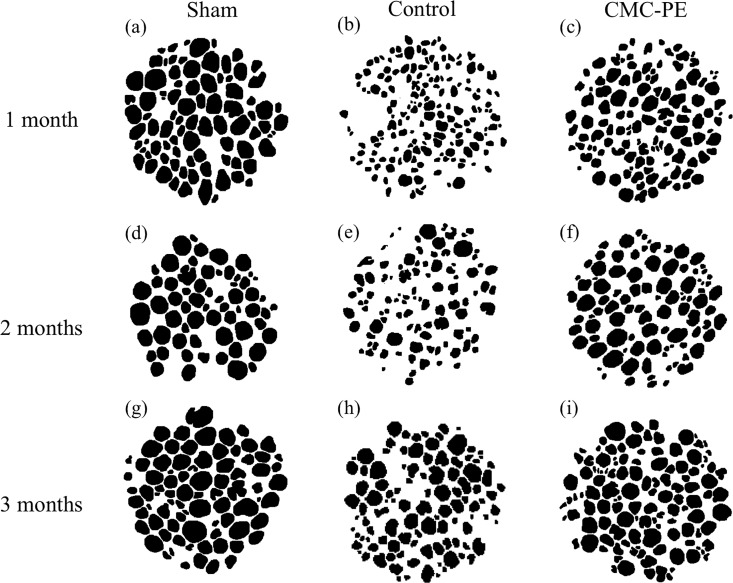
Typical examples of binary images in each group. Magnification, 40×; large diameter axons are evident at all time points in the sham group (a, b, c). Axons became repaired in stages in the control group (b, e, h) and obviously improved at early period (c) and then slowed (f, i) in the CMC-PE group.

Fig 8 shows typical axon morphology found in the ROIs. Binary images of the control group show that most axons survived and started to recover appreciably after two months, although the axons became significantly thinner during the three months of compression. This finding is consistent with the changes in the relative wet weight of the tibialis anterior muscle in the control group, indicating that simple nerve decompression can lead to significant functional recovery within three months in our animal model. On the other hand, many thick axons were obvious in the CMC-PE group even at one month, and they became more abundant at a much faster pace than in the control group throughout the experimental period. These results suggest that applying the CMC-PE hydrogel during nerve decompression and Improvement of nerve gliding significantly enhanced the structural and functional recovery of damaged axons in our animal model.

**Fig 9:**
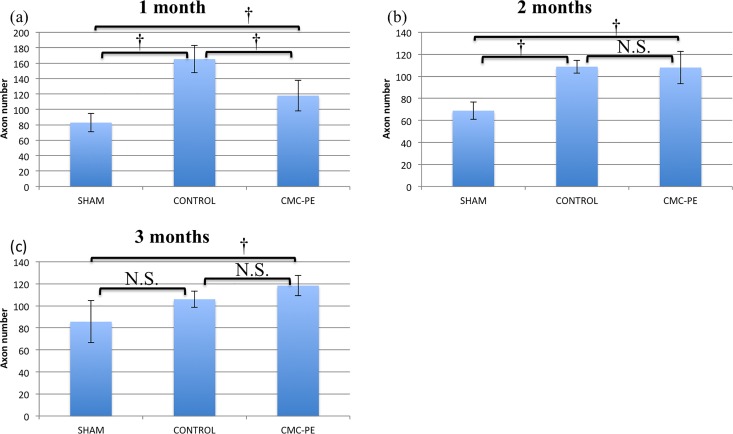
Evaluation of numbers of axons after nerve decompression. Number of axons significantly differed between CMC-PE and control groups at one (a), but not at two (b) and three (c) months. *P < 0.05, †P< 0.01.

**Fig 10:**
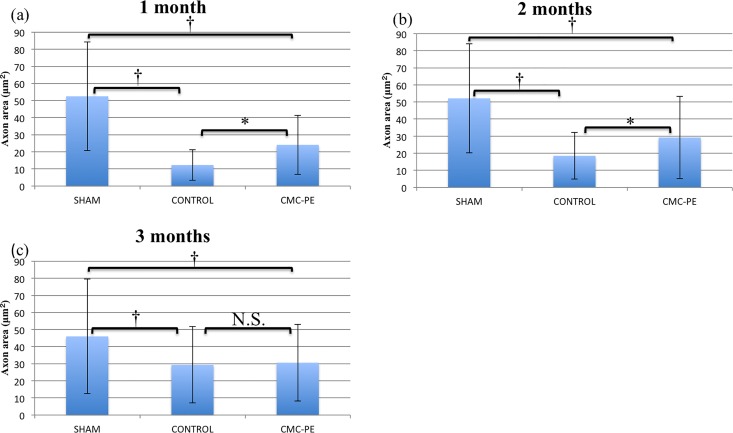
Evaluation of axon area after nerve decompression. Axon area was twice as large in the CMC-PE than in the control group at one (a) month. Significant differences at one (a) and two (b), but not at three months (c). *P < 0.05, †P< 0.01.

To confirm this, we quantitatively analyzed the axons using ImageJ Fig 9. The axon counts in one ROI each from the sham, control and CMC-PE groups at one, two and three months after nerve decompression were 82.8 ± 11.7, 165.2 ± 17.6 and 117.8 ± 19.8, 68.8 ± 7.8, 108.0 ± 5.9 and 108.8 ± 14.7 and 85.6 ± 19.1, 106.0 ± 7.3 and 118.4 ± 9.1, respectively. The control group probably contained more axons in a ROI of 100 μm, especially at one month due to severe axonal atrophy. However, the differences in axon counts were statistically significant only at one month after decompression. The axon areas in the sham, control and CMC-PE groups at one, two and three months after nerve decompression were 52.5 ± 31.8, 12.3 ± 9.0 and 24.1 ± 17.3, 52.1 ± 31.9, 18.5 ± 13.6 and 29.2 ± 24.0 and 46.0 ± 33.5, 29.4 ± 22.5 and 30.6 ± 22.4 μm^2^, respectively Fig 10. Unlike the axon counts, the axon area significantly differed between the control and CMC-PE groups at one and two months after decompression. The difference gradually decreased and lost statistical significance at three months. These findings showed that the CMC-PE gel enhanced axonal recovery after chronic nerve compression by reducing mechanical stress caused by postoperative scarring.

## Discussion

Chemical compounds have recently been applied to prevent postoperative scarring. Sodium hyaluronan, a polyglycan ester gel, pure alginate solution and autocross-linked polysaccharide gels have all shown promise for preventing postoperative nerve fibrosis in animal models [2,17,20,21,22,23]. However, the models in these experiments were all those of acute nerve injury and the effects of treatment in a model of chronic nerve damage have not been determined.

Chronic nerve compression neuropathies (entrapment neuropathies) are the most prevalent type of neuropathies in the peripheral nervous system and they usually occur at anatomical sites where nerves can be trapped by surrounding tissues. Although the word “compression” forms part of the description, the pathophysiology is more complicated for chronic than for acute nerve compression neuropathies. Millesi has described that the median nerve is drawn into the carpal tunnel by 9.6mm when the fingers were extended with dorsiflexion of the wrist [24]. If the nerve was compressed, this nerve gliding will be inhibited and cause nerve strain. Watanabe has shown that repeated small nerve strain makes more histologic, electrophysiologic or functional abnormalities than continuous nerve stretching [25]. Repeated external pressure as well as strain on the nerve hampers circulation in the vessels supplying the nerve, resulting in transient conduction blocks. As compression severity increases over time, structural changes such as scarring both in the perineurial and endoneurial structures, axonal stenosis and demyelination arise and many axons degenerate and become replaced by scar tissue at the advanced stages [26].

Peripheral nerves have an adventitial tissue called the paraneurium that allows for movement between nerves and surrounding tissues [27]. According to Millesi who classified different degrees of fibrosis in peripheral nerves with reference to neurolysis procedures [28], internal neurolysis to remove fibrotic tissue inside a nerve trunk can have deleterious effects because it not only impedes the blood supply to nerve fascicles but also induces an enhanced post-surgical fibrotic response. Fibrosis of the paraneurium that remains outside the epineurium still notably results in the same consequences as fibrosis of the epineurium [29]. In this context, therapy with anti-adhesion molecules can be beneficial as an adjuvant treatment for all types of neurolysis.

Here, we tested the hypothesis that the novel anti-adhesive CMC-PE hydrogel is a useful adjuvant in the surgical treatment of entrapment neuropathies in an established and widely applied animal model of chronic nerve compression [11,12,13,14]. Mackinnon described that one of the initial change was localized then diffuse, demyelination occurred [30]. Our study has recognized axon area of the outer portions of nerve fascicles that tend to be more advanced localized demyelination. Biomechanical tests of our model showed that CMC-PE hydrogel exerted powerful anti-adhesive effects after surgical nerve decompression. Therefore, nerves in the CMC-PE group were probably exposed to significantly less mechanical stress than those in the control group. Excluded mechanical stress encouraged remyelination. Therefor, CMC-PE gel enhanced axonal recovery at one month. Morphometric analysis showed nerve remyelination. Buck up these results electrophysiological evaluation also showed enhanced nerve recovery. Impulses are conducted by salutatory conduction in myelinated fibers. And it is related to MCV. In addition, wet muscle weight showed enhancement of early recovery.

These results showed that anti-adhesive gels can be a valuable adjunct for simple nerve decompression. We previously showed that CMC-PE gel significantly inhibits fibrosis in perineural structures even after aggressive internal neurolysis [4]. Therefore, we speculate that the CMC-PE gel will also be useful for internal neurolysis.

## Conclusions

Our CMC-PE hydrogel prevented postoperative nerve adhesions and significantly enhanced morphological and physiological recovery in an animal model of chronic nerve compression neuropathy.

## References

[1] Sunderland S. The nerve lesion in the carpal tunnel syndrome. J. Neurol. Neurosurg Psychiatry. 1976; 39: 615–626. 993794 10.1136/jnnp.39.7.615 PMC492389

[2] Dam-Hieu P, Lacroix C, Said G, Devanz P, Liu S, Tadie M. Reduction of postoperative perineural adhesions by Hyaloglide gel: an experimental study in the rat sciatic nerve. Neurosurgery. 2005; 56: 425–433. 15794840 10.1227/01.neu.0000156845.41626.e9

[3] Mackinnon SE, Dellon AL. Experimental study of chronic nerve compression. Clinical implications. Hand Clin. 1986; 2: 639–650. 3793765

[4] Yamamoto M, Endo N, Ito M, Okui N, Koh S, Kaneko H, et al (2010). Novel polysaccharide-derived hydrogel prevents perineural adhesions in a rat model of sciatic nerve adhesion. J Orthop Res. 2010; 28: 284–288. 10.1002/jor.21004 19780191

[5] Yamamoto T, Narushima M, Yoshimatsu H, Yamamoto N, Mihara M, Koshima I. Free anterolateral thigh flap with vascularized lateral femoral cutaneous nerve for the treatment of neuroma-in-continuity and recurrent carpal tunnel syndrome after carpal tunnel release. Microsurgery. 2014; 34: 145–148 10.1002/micr.22135 23843323

[6] Xu J, Varitimidis SE, Fisher KJ, Tomaino MM, Sotereanos DG. The effect of wrapping scarred nerves with autogenous vein graft to treat recurrent chronic nerve compression. J Hand Surg Am. 2000; 25: 93–103. 10.1053/jhsu.2000.jhsu025a0093 10642478

[7] Okui N, Yamamoto M, Fukuhira Y, Kaneko H, Hirata H. A new nerve coaptation technique using a biodegradable honeycomb-patterned film. Microsurgery. 2012; 32: 466–474. 10.1002/micr.21998 22707444

[8] Dumanian GA, McClinton MA, Brushart TM. The effects of free fat grafts on the stiffness of the rat sciatic nerve and perineural scar. J Hand Surg Am. 1999; 24: 30–36. 10.1053/jhsu.1999.jhsu24a0030 10048513

[9] Abitbol JJ, Lincoln TL, Lind BI, Amiel D, Akeson WH, Garfin SR. A new experimental model. Spine. 1994; 19: 1809–1814. 7973979 10.1097/00007632-199408150-00004

[10] Ikeda K, Yamauchi D, Tomita K. Preliminary study for prevention of neural adhesion using an absorbable oxidised regenerated cellulose sheet. Hand Surg. 2002; 7: 11–14. 12365044 10.1142/s0218810402000911

[11] O'Brien JP, Mackinnon SE, MacLean AR, Hudson AR, Dellon AL, Hunter DA. A model of chronic nerve compression in the rat. Ann Plast Surg. 1987; 19: 430–435. 3688790 10.1097/00000637-198711000-00008

[12] Gupta R, Rowshan K, Chao T, Mozaffar T, Steward O. Chronic nerve compression induces local demyelination and remyelination in a rat model of carpal tunnel syndrome. Exp. Neurol. 2004; 187: 500–508. 10.1016/j.expneurol.2004.02.009 15144876

[13] Gupta R, Nassiri N, Hazel A, Bathen M, Mozaffar T. Chronic nerve compression alters Schwann cell myelin architecture in a murine model. Muscle Nerve 2012; 45: 231–241. 10.1002/mus.22276 22246880 PMC3262776

[14] Pham K, Nassiri N, Gupta R. c-Jun, krox-20, and integrin beta4 expression following chronic nerve compression injury. Neurosci Lett. 2009; 465: 194–198. 10.1016/j.neulet.2009.09.014 19765400 PMC3262774

[15] Iwatsuki K, Arai T, Ota H, Kato S, Natsume T, Kurimoto S, et al Targeting anti-inflammatory treatment can ameliorate injury-induced neuropathic pain. PLoS One. 2013; 8(2): e57721 10.1371/journal.pone.0057721 23469058 PMC3585184

[16] Okui N, Yamamoto M, Fukuhira Y, Kaneko H, Hirata H. Artificial perineurium to enhance nerve recovery from damage after neurolysis. Muscle Nerve. 2010; 42:570–575. 10.1002/mus.21727 20878739

[17] Ohsumi H, Hirata H, Nagakura T, Tsujii M, Sugimoto T, Miyamoto K, et al Enhancement of perineurial repair and inhibition of nerve adhesion by viscous injectable pure alginate sol. Plast Reconstr Surg. 2005; 116: 823–830. 16141822 10.1097/01.prs.0000176893.44656.8e

[18] Okui N, Yamamoto M, Fukuhira Y, Kaneko H, Hirata H. Artificial perineurium to enhance nerve recovery from damage after neurolysis. Muscle Nerve. 2010; 42: 570–575. 10.1002/mus.21727 20878739

[19] Aguayo A, Nair CPV, Midgley R. Experimental progressive compersion neuropathy in the rabbit: histologic and electrophysiologic studies. Arch Neurol. 1971; 24: 358–364. 4323377 10.1001/archneur.1971.00480340090010

[20] Ikeda K, Yamauchi D, Osamura N, Hagiwara N, Tomita K. Hyaluronic acid prevents peripheral nerve adhesion. Br J Plast Surg. 2010; 56: 342–347. 10.1016/s0007-1226(03)00197-8 12873461

[21] McCall TD, Grant GA, Britz GW, Goodkin R, Kliot M. Treatment of recurrent peripheral nerve entrapment problems: role of scar formation and its possible treatment. Neurosurg. Clin N Am. 2001; 12: 329–339. 11525211

[22] Isla A, Martinez JR, Perez-Lopez C, Perez Conde C, Morales C, Budke M. A reservable antiadhesion barrier gel reduces the perineural adhesions in rats after anastomosis. J Neurosurg Sci. 2003; 47: 195–199. 14978473

[23] Petersen J, Russell L, Andrus K, Mackinnon M, Silver J, Kliot M. Reduction of extraneural scarring by ADCON-T/N after surgical intervention. Neurosurgery. 1996; 38: 976–983. 8727824 10.1097/00006123-199605000-00025

[24] Miillesi H, Zoch G, Rath th. The gliding apparatus of peripheral nerve and its clinical significance. Ann Hnad Surg. 1990; 9:87–97 10.1016/s0753-9053(05)80485-5 1695518

[25] Watanabe M, Yamaga M, Kato T, Ide J, Kitamura T, Takagi K. The implication of repeated versus continuous strain on nerve function in a rat forelimb model. J Hand Surg. 2001; 26A: 663–669 10.1053/jhsu.2001.24142 11466641

[26] Nishimura T, Hirata H, Tsuji M, Iida R, Hoki Y, Iino T, et al Pathomechanism of entrapment neuropathy in diabetic and nondiabetic rats reared in wire cages. Histol Histopathol. 2008; 23: 157–66. 17999372 10.14670/HH-23.157

[27] Pho RWH, Meyer VE. Microsurgical technique in orthopaedics Butterworths,. London 1988

[28] Millesi H, Rath TH, Reihsner R, Zoch G. Microsurgical neurolysis: Its anatomical and physiological basis and its classification. Microsurgery. 1993; 14: 430–439. 8264374 10.1002/micr.1920140703

[29] Mazal PR, Millesi H. Neurolysis: Is it beneficial or harmful? Acta Neurochir. 2005; 92: 3–6. 10.1007/3-211-27458-8_1 15830957

[30] Mackinnon SE. Pathophysiology of nerve compression. Hand Clin. 2002; 18: 231–241 12371026 10.1016/s0749-0712(01)00012-9

